# Core Self-Evaluation and Burnout among Nurses: The Mediating Role of Coping Styles

**DOI:** 10.1371/journal.pone.0115799

**Published:** 2014-12-26

**Authors:** Xiaofei Li, Lili Guan, Hui Chang, Bo Zhang

**Affiliations:** 1 Emergency Department, The First Affiliated Hospital of China Medical University, Shenyang, China; 2 Faculty of Nursing, Huaiyin Advanced Vocational and Technical School of Health, Huaian, China; 3 Social Education Department, Center for Health Education of Liaoning Province, Shenyang, China; 4 Nursing Department, The First Affiliated Hospital of China Medical University, Shenyang, China; University of Geneva, Switzerland

## Abstract

**Objectives:**

This study aimed to determine the potential association between core self-evaluation and the burnout syndrome among Chinese nurses, and the mediating role of coping styles in this relationship.

**Methods:**

A cross-sectional survey was conducted in Shenyang, China, from May to July, 2013. A questionnaire which consisted of the Maslach Burnout Inventory-General Survey (MBI-GS), the Core Self-Evaluation Scale (CSE), and the Simplified Coping Style Questionnaire (CSQ), was completed by a total of 1,559 nurses. Hierarchical linear regression analyses and the Sobel test were performed to determine the mediating role of coping styles on the relationship between CSE and burnout.

**Results:**

Nurses who had higher self-evaluation characteristics, reported less emotional exhaustion and cynicism, and higher professional efficacy. Coping style had a partial mediating effect on the relationship between CSE and the burnout syndrome among nurses.

**Conclusions:**

Core self-evaluation had effects on burnout and coping style was a mediating factor in this relationship among Chinese nurses. Therefore, the improvement of coping strategies may be helpful in the prevention of burnout among nurses, thus enhancing professional performance.

## Background

Burnout is the result of continuous work pressures that are not effectively handled [1] and is defined by three dimensions: emotional exhaustion (EE), cynicism (CY) and reduced professional efficacy (PE). Emotional exhaustion refers to an individual being overextended emotionally, which leads to the depletion of an individual's resources. Cynicism refers to a negative, callous or excessively detached response to various aspects of the job. Reduced professional efficacy represents a general sense of one's inefficacy at work and to a feeling of lack of professional success and competency [2]. Burnout has been recognized as an occupational hazard in various people-oriented professions [3]. In the area of healthcare, nursing staff face high demands in terms of quality services, and are subjected to numerous stressful situations. Therefore, nurses are at risk of burnout [4]. A severe nursing shortage and an increase in demanding workload in terms of physical, emotional and moral stress, combined with comparatively low wages and devaluation of the profession have caused nurses to experience burnout in China. Burnout among nurses can result in mental fatigue, anxiety, lack of motivation, and absence from work [5], which undoubtedly threatens not only their own health, but also that of their patients. Therefore, the prevention of burnout among nurses is essential for improving the quality of patient care.

With regard to personal resources, traditional research on personality and burnout has focused on the Big Five personality dimensions and emotional affectivity [6], [7]. However, recently, Core self-evaluation (CSE) has received a great deal of attention in personality research. CSE, a broad personality concept, which describes an individual's evaluation about themselves, their ability, and their control, consists of four traits: self-esteem, general self-efficacy, locus of control, and neuroticism [8]. Numerous studies have reported that CSE significantly influenced salary [9], goal setting [10], job satisfaction [11], job performance [10], [12], career commitment [13], and job burnout [13], [14]. In addition, Spence Laschinger et al. identified the important role of CSE on nurse managers' burnout [15]. However, previous studies did not determine how CSE influences burnout.

Stress is accompanied by the process of coping [16]. Coping styles are defined as stable psychological and behavioral strategies to overcome or tolerate external and internal challenges or stressors [17]. Some people cope with stress actively, while others cope passively. Active coping strategies are either behavioral or psychological responses designed to change the nature of the stressor itself or how one thinks about it, whereas passive coping strategies lead people into activities (such as alcohol use) or mental states (such as withdrawal) which prevent them from directly addressing stressful events [18]. Coping behaviors of individuals contribute to the explanation of why exposure to the same stressors may cause burnout in some subjects, but not in others [19]. A study conducted in Hong Kong by Wang et al. indicated that making use of effective coping strategies plays a pivotal role in reducing stress among nurses [20]. The coping strategies used by nurses may vary with respect to their personal, psychological, and cultural factors [21]. A previous study reported that Chinese nurses tended to cope actively with stress associated with decreased personal achievement, but passively when the stress originated from resource and environment problems, patient care and interaction issues, and from interpersonal relationships and management issues [22], suggesting regional differences in the coping styles of nurses. According to previous studies, burnout can be alleviated by coping strategies. Active coping may protect against the negative impact of stressors on adaptive outcomes by strengthening the person's coping efficacy in a specific situation [4]. However, Payne et al. [23] concluded that the investigation of problem-focused and emotion-focused coping in relation to burnout, oversimplied the coping-burnout relationship in a sample of hospice nurses. The association between CSE and burnout and the association between coping styles and burnout have been investigated in previous studies [5], [13], [14], [15], [23]. In addition, Kammeyer-Mueller et al. [24] investigated the role of CSE in the coping process, and demonstrated that individuals with high CSE practice less avoidance coping. However, to our knowledge, there has been no integrative effort to examine coping styles as a mediator in the relationship between CSE and burnout.

Nurses need to care for themselves before they can provide care for their clients. Given the high stress, difference in coping styles, as well as high workload in Chinese nurses, there is a need to develop an educational program and strategies for the prevention of burnout among nurses. For this purpose, it is important to clarify the relationships between burnout, CSE, and coping styles among Chinese nurses. In the present study, we examined the relationship between CSE and burnout and determined the mediating role of coping styles on the relationship between CSE and burnout using a cross-sectional survey of 1,559 Chinese nurses from Shenyang, China.

## Methods

### Participants and procedure

A cross-sectional survey was conducted in Shenyang, China from May to July, 2013. Respondents in this study were selected from five hospitals which included three university-affiliated hospitals, one provincial tertiary hospital, and one municipal tertiary hospital. The inclusion criteria required that an individual held the RN licensure granted by the Ministry of Health, PR China and was currently working in one of the hospitals as a registered nurse. Head nurses and administrators were excluded. Anonymous questionnaires were distributed and collected during staff meetings in all hospital units by the researcher (the third author) over a 4-week period. A sample of 1995 clinical nurses was recruited and 1,662 questionnaires were returned (return rate: 83.3%). 103 questionnaires were discarded due to incomplete data, resulting in 1,559 analyzed questionnaires.

Ethical approval of the study was given by the medical ethics committee of China Medical University. All participants signed an informed consent form.

### Instruments

#### Core Self-Evaluation Scale (CSE)

This scale was developed by Judge et al. [8] and is a 12-item self-report measure of CSE. Items are rated from 1 (strongly disagree) to 5 (strongly agree). The scale scores are the sum of the ratings of the items. Relevant items were reverse-coded. The Chinese version of the CSE has previously been used in the Chinese population and demonstrated good reliability and validity [25]. In this study, the Cronbach's alpha coefficient for CSE was 0.745.

#### Maslach Burnout Inventory–General Survey (MBI-GS)

The MBI-GS was developed by Maslach et al. [26], [27] and is a 15-item self-report measure of job burnout which includes three dimensions, emotional exhaustion, cynicism, and professional efficacy. The items are scored on a Likert scale from 0 (never) to 6 (every day). Higher scores on emotional exhaustion and cynicism dimensions and lower scores on professional efficacy dimension indicated higher levels of burnout. The Chinese version of the MBI has been widely used in Chinese studies and has demonstrated satisfactory reliability and validity [28], [29]. In the present study, Cronbach's alpha coefficients for the three dimensions of MBI-GS were 0.896, 0.747, and 0.825, respectively.

#### The Simplified Coping Style Questionnaire (CSQ)

This questionnaire developed by Xie YN [30] was based on the Ways of Coping questionnaire by Folkman and Lazarus [31] and is a 20-item self-report that includes two dimensions, active coping (12-item) and passive coping (8-item). The items were measured using four-point Likert scales (0 =  never; 3 =  very often). The instrument has been commonly used in China and the internal consistency measured by Cronbach's alpha was reported to be 0.78 [30]. In this study, the Cronbach's alpha coefficients for the two dimensions of SCSQ were 0.796 and 0.728, respectively.

#### Demographic data sheet

Demographic data including gender, age, educational level, work experience, and job rank were obtained from a structured questionnaire.“Educational level”was categorized as “high school or under”, “junior college” and “undergraduate or above”. “job rank” was categorized as “junior nurse”, “senior nurse” and “nurse-in-charge”.

### Statistics

Data were imported to the Epidata 3.1 database. All analyses were conducted using SPSS 17.0 for Windows and all statistical tests were two-sided (α = 0.05). The distributions of dimensions of burnout in categorical demographic characteristics were tested by one-way ANOVA. Pearson correlation was performed to test the relationship between core self-evaluation, coping styles, and burnout. Baron and Kenny's [32] technique was used to test the mediating effect of coping styles on the relationship between core self-evaluation and burnout. According to Baron and Kenny [32], the following conditions should be satisfied to establish mediation: (1) The independent variable (CSE) is significantly associated with the dependent variable (Emotional exhaustion/Cynicism/Professional efficacy), (2) the independent variable (CSE) is significantly associated with the mediator (Active coping/Passive coping), and (3) the mediator (Active coping/Passive coping) is significantly associated with the dependent variable (Emotional exhaustion/Cynicism/Professional efficacy), and the effect of the independent variable (CSE) on the dependent variable (Emotional exhaustion/Cynicism/Professional efficacy) reduces when the mediator (Active coping/Passive coping) is added to the model (partial mediator). If the independent variable does not affect the dependent variable when the mediator is added to the model, then full mediation is established.

Before performing the regression analyses, all the continuous variables were centered in order to avoid multicollinearity [33]. In addition, tolerance and the variance inflation factor were used to check for multicollinearity. We performed Pearson correlation and one hierarchical linear regression analysis for each of the three burnout dimensions to test the mediating effect. In step one of the hierarchical linear regression analyses, the control variables and positive results of variance analysis were added to the model. In the present study, we included gender, age, educational level, work experience and job rank in the model as potential confounders. Because educational level and job rank are categorical variables without a linear trend, dummy variables for these two variables were set. For educational level, “High school or under” was set as the reference group. For job rank, “junior nurse” was set as the reference group. In step 2, core self-evaluation was added. In step 3, active coping and passive coping were added.

In addition, the statistical significance of the mediation effect was confirmed using the Sobel test.

## Results

The demographic and working characteristics of the subjects and the distribution of each dimension of burnout in the categorical items are shown in Table 1. Mean emotional exhaustion, cynicism, and professional efficacy differed across the age and work experience groups. Nurses aged 30–40 years had the highest scores in emotional exhaustion and cynicism and nurses aged over 40 years had the highest scores in professional efficacy. The group with 10–15 years work experience had the highest scores in the three dimensions of burnout. Mean emotional exhaustion and cynicism differed between male and female nurses. Female nurses were more easily affected by emotional exhaustion and cynicism. Mean emotional exhaustion and professional efficacy differed among the job rank groups. Senior nurses had the highest scores in emotional exhaustion, whereas nurse-in-charge had the highest scores in professional efficacy. In addition, mean professional efficacy differed across the education level groups. Nurses with an undergraduate degree or above had the highest scores in professional efficacy.

**Table 1:** Demographics and working variables of the subjects and distribution of the MBI-GS.

Variable	N(%)	Mean(SD)		
		Emotional exhaustion	Cynicism	Professional efficacy
Gender		*P* = 0.011	*P* = 0.010	*P* = 0.161
Male	60(3.8%)	11.58(7.19)	7.57(5.06)	24.42(9.58)
Female	1499(96.2%)	14.28(8.05)	9.67(6.20)	25.90(7.95)
Age (yr)		*P* = 0.011	*P* = 0.037	*P* = 0.004
<30	1027(65.9%)	14.41(7.81)	9.46(6.00)	25.39(8.02)
30–40	334(21.4%)	14.42(8.27)	10.31(6.60)	26.42(7.37)
>40	198(12.7%)	12.58(8.60)	9.03(6.27)	27.21(8.87)
Education level		*P* = 0.210	*P* = 0.099	*P* = 0.007
High school or under	213(13.7%)	13.29(7.67)	9.08(5.94)	24.93(8.68)
Junior college	840(53.9%)	14.37(7.83)	9.44(6.00)	25.54(7.87)
Undergraduate or above	506(32.5%)	14.23(8.49)	10.04(6.54)	26.71(7.91)
Work experience(years)		*P* = 0.000	*P* = 0.026	*P* = 0.003
<5	862 (55.2%)	14.10(7.64)	9.35(5.85)	25.18(8.06)
5–10	326 (20.9%)	15.14(8.41)	9.99(6.55)	26.30(7.22)
10–15	88 (5.6%)	15.92(7.54)	11.22(6.59)	27.05(6.26)
>15	283 (18.2%)	12.75(8.67)	9.34(6.48)	26.94(8.65)
Job rank		*P* = 0.029	*P* = 0.209	*P* = 0.000
Junior nurse	756 (48.5%)	13.98(7.57)	9.31(5.78)	24.83(8.26)
Senior nurse	533 (34.2%)	14.86(8.17)	9.81(6.43)	26.72(7.15)
Nurse-in-charge	270 (17.3%)	13.36(8.91)	9.94(6.71)	26.93(8.62)

### Pearson correlations

The results of Pearson correlation analysis among core self-evaluation, coping styles, and burnout are shown in Table 2. The CSE scale was significantly correlated with the dimensions of burnout (*r = −*0.343, −0.345, 0.282, *P*<0.01), where nurses with a higher score on the CSE scale had better professional efficacy, whereas nurses with a lower score on the CSE tended to be more susceptible to emotional exhaustion and cynicism. In addition, CSE was significantly associated with the dimensions of coping styles (*r = *0.314, −0.254, *P*<0.01), where nurses with active coping styles were found to have a higher score on the CSE scale and passive coping styles had a negative impact on the CSE score.

**Table 2:** Means, standard deviations (SD) and correlations of all variables.

Variables	Mean	SD	1	2	3	4	5	6
1 Emotional exhaustion	14.18	8.03	1					
2 Cynicism	9.59	6.18	0.704**	1				
3 Professional efficacy	25.84	8.02	0.027	0.001	1			
4 Core self-evaluation	40.47	5.34	−0.343**	−0.345**	0.282**	1		
5 Active coping	24.11	5.38	−0.156**	−0.144**	0.271**	0.314**	1	
6 Passive coping	9.96	4.13	0.206**	0.239**	−0.095**	−0.254**	0.171**	1

*P<0.05, **P<0.01 (two-tailed).

### The mediating role of coping styles on the relationship between CSE and emotional exhaustion

As shown in Table 3, both CSE score and active coping style were negatively associated with emotional exhaustion (*β* = −0.500 and −0.148, respectively, *P*<0.01), the higher the score on the CSE scale and the more active coping style a nursing professional had, the lower the chance of emotional exhaustion. Conversely, a positive correlation between emotional exhaustion and passive coping style was observed in our subjects (*β* = 0.308, *P*<0.01). Importantly, coping styles (Active coping/Passive coping) had a partial mediating effect on the relationship between CSE and emotional exhaustion, in that the regression coefficient for CSE was reduced when coping styles were added to the model (from *β* = 0.500 to *β* = 0.396). The result of the Sobel test confirmed the significance of the mediating effect of active coping (z = −5.66, *P*<0.001) and passive coping (z = −6.44, *P*<0.001). In addition, gender, age, and job rank affected emotional exhaustion.

**Table 3:** Results of hierarchical linear regression analyses, with emotional exhaustion as the criterion variable.

Variables	Emotional Exhaustion		
	Step 1(*β*)	Step 2(*β*)	Step 3(*β*)
Gender	2.745**	2.628**	2.812**
Age	−0.316**	−0.265**	−0.273**
Work experience(years)	0.116	0.096	0.103
Job rank-1	1.623**	1.675**	1.847**
Job rank-2	2.454*	2.384*	2.718**
Core self-evaluation		−0.500**	−0.396**
Active coping			−0.148**
Passive coping			0.308**
R2	0.020	0.134	0.158
Δ R2	0.020	0.114	0.024

Note: Job rank-1 indicates “senior nurse” vs. “junior nurse”, Job rank-2 indicates “nurse-in-charge” vs. “junior nurse”.

*P<0.05, **P<0.01 (two-tailed).

### The mediating role of coping styles on the relationship between CSE and cynicism

As shown in Table 4, both CSE and active coping style were negatively associated with cynicism (*β* = −0.390 and −0.107, respectively, *P*<0.01). However, negative coping style was positively associated with cynicism (*β* = 0.288, *P*<0.01). Coping style not only directly impacted the cynicism score, but also affected it indirectly by partially mediating the relationship between CSE and cynicism. The regression coefficient for CSE in relation to cynicism score was reduced from 0.39 to 0.303 (*P*<0.01) when coping styles were added to the regression analysis. The result of the Sobel test supported the significance of the mediating effect of active coping (z = −5.26, *P*<0.001) and passive coping (z = −7.00, *P*<0.001). In addition, gender, age, and work experience also affected cynicism.

**Table 4:** Results of hierarchical linear regression analyses, with cynicism as the criterion variable.

Variables	Cynicism		
	Step 1(*β*)	Step 2(*β*)	Step 3(*β*)
Gender	2.082*	1.998**	2.194**
Age	−0.195*	−0.155*	−0.159*
Work experience(years)	0.167*	0.149*	0.164*
Core self-evaluation		−0.390**	−0.303**
Active coping			−0.107**
Passive coping			0.288**
R2	0.008	0.126	0.159
Δ R2	0.008	0.117	0.033

*P<0.05, **P<0.01 (two-tailed).

### The mediating role of coping styles on the relationship between CSE and professional efficacy

The impact of coping styles on professional efficacy is shown in Table 5. Both CSE and active coping style were positively associated with professional efficacy (*β* =  0.413 and 0.329, respectively, *P* <0.01). However, passive coping style was negatively associated with professional efficacy (*β* =  −0.148, *P* <0.01). More importantly, coping styles (active coping/passive coping) indirectly affected professional efficacy by partially mediating the relationship between CSE and professional efficacy, in that the regression coefficient for CSE decreased when active coping/passive coping was added to the regression analysis (from *β* = 0.413 to *β* = 0.283, *P*<0.01). The result of the Sobel test supported the significance of the mediating effect of active coping (z = 8.47, *P*<0.001) and passive coping (z = 3.54, *P*<0.001).

**Table 5:** Results of hierarchical linear regression analyses, with professional efficacy as the criterion variable.

Variables	Professional efficacy		
	Step 1(*β*)	Step 2(*β*)	Step 3(*β*)
Age	−0.153	−0.189	−0.180
Work experience(years)	0.126	0.131	−0.131
Education-1	0.537	0.664	0.767
Education-2	0.935	1.161	1.272
Job rank-1	1.882**	1.856**	1.673**
Job rank-2	1.984	2.061	1.533
Core self-evaluation		0.413**	0.283**
Active coping			0.329**
Passive coping			−0.148**
R2	0.018	0.096	0.136
Δ R2	0.018	0.078	0.041

Note: Job rank-1 indicates “senior nurse” vs. “junior nurse”, Job rank-2 indicates “nurse-in-charge” vs. “senior nurse”. Education-1 indicates “Junior college” vs. “High school or under”, Education-2 indicates “Undergraduate or above” vs. “High school or under”.

*P<0.05,

**P<0.01 (two-tailed).

## Discussion

This study investigated the relationship between CSE, coping styles, and burnout dimensions among Chinese nurses, and demonstrated that CSE was an integrated personality variable that may affect job burnout, and coping styles might have both a direct and indirect effect on burnout. Our data suggest that strategies which promote active coping styles among nursing professionals may help to reduce job burnout, and thus enhance nursing efficacy. With regard to the relationship between CSE and burnout, CSE was shown by our study and others [13], [14] to be a possible predictor of job burnout. This was supported by our data which showed that nurses with higher CSE scores had less emotional exhaustion and cynicism and higher professional efficacy. CSE is a deep personality trait. Those with higher CSE scores will always have higher self-confidence and esteem, and a more positive attributive style [13], which may result in lower levels of emotional exhaustion and cynicism. CSE reflects the long-term faith of individuals in their ability to maintain a stable self and a sense of control, which are important in the evaluation of individual ability [34], [35]. Thus, those with a higher CSE score might have a higher level of professional efficacy.

Positive factors relating to the prevention of nursing burnout such as hardiness, active coping, and social support were found to be the most important buffering factors [4]. In the present study, active coping was negatively associated with emotional exhaustion and cynicism, and was positively associated with professional efficacy. These findings contribute to the understanding that coping styles have a significant effect on burnout, and active coping may be a positive resource for combating burnout.

As shown in Table 3, as passive coping increased, emotional exhaustion scores increased accordingly. This is consistent with Gibbons' study [36], where active coping and older age played a clear role in decreasing vulnerability to emotional exhaustion and cynicism. It appears that younger nurses experience more burnout than their older colleagues. This may indicate that with increased life experience, individuals might have a lower tendency to use passive coping styles and more confidence, thus have less burnout. Active coping is helpful in preventing cynicism, and this is in agreement with a previous study which found that active coping style can decrease the negative impact of stressors by strengthening one's coping efficacy and dynamics in a specific situation [4]. Dysfunctional coping strategies in nurses results in the deterioration of nurse-patient relationships, and failures are increasingly experienced leading to a gradual sense of lack of personal accomplishment [4]. Nurses who usually face disease, death and other situations tend to develop emotionally-negative characteristics, and feelings of emotional exhaustion. If no effective resources are adopted to cope with exhaustion, this will lead to cynicism and reduced professional efficacy. Conversely, when a successful coping style is adopted (e.g., active coping), nurses can achieve their goals and professional efficacy improves. Moreover, Folkman and Lazarus [31] stated that the problem-solving coping style can result in an improvement in the person-environment relationship, thus achieving a better cognitive appraisal and a more positive emotional response. An optimistic coping strategy can result in nurses using problem solving to deal with their difficulties to effectively reduce their stress levels [37].

Our study revealed that coping styles (Active coping/Passive coping) partially mediated the effects of CSE on emotional exhaustion, cynicism and professional efficacy. Nurses with lower CSE might be more likely to use passive coping which in turn leads to higher levels of emotional exhaustion and cynicism, and lower levels of professional efficacy. Compared to changing the CSE score in nurses, it might be more positive and feasible for hospitals to conduct nurse training on problem-solving skills with the goal of helping nurses develop and employ active coping styles to deal with the stress they face at work. This type of training would be expected to improve nurses' health and well-being and to reduce professional burnout, and thus improve the quality of healthcare.

Although the present study demonstrates the important role of self-evaluation and coping styles on job burnout among Chinese nursing professionals, there are limitations regarding the design of the study. Firstly, we conducted this study in large general hospitals, which may not necessarily reflect the burnout experienced by nurses in different hospitals and community health centers. Secondly, the study relied only on self-report measures which may have introduced bias. Therefore, future studies in different hospitals and consisting of more objective parameters, such as behavioral and physiological indicators, as well as broader influencing factors, will provide more insightful knowledge regarding the inter-relationships between self-evaluation, coping styles, and nursing burnout. In addition, this was a cross-sectional study, thus the causality could not been established in this study. Prospective studies should be conducted in the future to confirm the findings obtained in this study.

## Conclusions

The present study demonstrated that CSE may be a possible predictor of job burnout among nursing professionals, indicating that nurses with a high CSE score display less burnout. More importantly, coping style might be an important factor which can affect burnout directly or indirectly by partially mediating the relationship between CSE and job burnout. Our results suggest that when active coping strategies are adopted, burnout is likely to be reduced. Therefore, interventions focused on the improvement of coping strategies may be helpful in the prevention of job burnout in nurses, thus enhancing nursing care efficacy.

## Supporting Information

S1 Table **The clinical data of 1559 nurses, which were analyzed in this study.**
(XLS)
